# Malignant cerebral edema after endovascular thrombectomy: a multimodal prediction model based on post-thrombectomy cerebral hyperdensity and natural language processing

**DOI:** 10.3389/fmed.2026.1857047

**Published:** 2026-08-05

**Authors:** Guolan Song, Jiahong Fu, Yuhan Chen, Yujie Shen, Jiayi Hong, Feifan Liu, Shiying Gai, Huan Liu, Dekuai Tong, Song Cheng, Jun Han, Jingjing Fu, Jian Ding

**Affiliations:** 1 Department of Radiology, The Fourth Affiliated Hospital of School of Medicine, International School of Medicine, International Institutes of Medicine, Zhejiang University, Yiwu, Zhejiang, China; 2 Department of Neurology, The Fourth Affiliated Hospital of School of Medicine, International School of Medicine, International Institutes of Medicine, Zhejiang University, Yiwu, Zhejiang, China; 3 Department of Neurointervention, The Fourth Affiliated Hospital of School of Medicine, International School of Medicine, International Institutes of Medicine, Zhejiang University, Yiwu, Zhejiang, China; 4 Department of Radiology, The First Hospital of Jiaxing, The Affiliated Hospital of Jiaxing University, Jiaxing, Zhejiang, China; 5 Department of Radiology, Tongde Hospital of Zhejiang Province, Hangzhou, Zhejiang, China

**Keywords:** deep learning, endovascular thrombectomy, malignant cerebral edema, multimodal fusion, natural language processing

## Abstract

**Background:**

Early prediction of malignant cerebral edema (MCE) following endovascular thrombectomy (EVT) is critical for guiding timely interventions. This study aimed to develop and validate a multimodal prediction, integrating non-contrast CT (NCCT) features and natural language processing (NLP)-encoded clinical data to predict MCE after EVT.

**Methods:**

In this multi-center retrospective study, 373 patients treated with EVT were included, comprising internal (*n* = 287) and external (*n* = 86) cohorts. MCE was defined as a midline shift of ≥5 mm. Deep imaging features were extracted using a ResNet-101 model, the NCCT slice demonstrating the maximal extent of post-thrombectomy cerebral hyperdensity (PCHD). Concurrently, a pre-trained NLP model, BioClinicalBERT, was utilized to generate semantic embeddings from synthesized clinical narratives derived from standard admission variables. A multimodal fusion model integrating these features was subsequently evaluated against single-modality models and the diagnostic performance of human experts.

**Results:**

In the independent external cohort, the multimodal fusion model achieved an area under the receiver operating characteristic curve (AUC) of 0.800 [95% confidence interval (CI): 0.700–0.901] and an accuracy of 80.2%, demonstrating superior performance compared to clinical-only (AUC = 0.654), ResNet-only (AUC = 0.707), and BERT-only (AUC = 0.560) models. SHapley Additive exPlanations (SHAP) analysis revealed NLP-derived semantic features as the principal predictors. Furthermore, AI assistance improved the diagnostic performance of senior neuroradiologists (AUC: 0.709–0.763; *p* < 0.05) and increased their specificity increased from (78.1% to 84.4%).

**Conclusion:**

A multimodal framework integrating targeted NCCT imaging features with NLP-encoded clinical data yields an accurate multimodal tool for early MCE prediction. This multimodal approach enhances human decision-making in emergency workflows.

## 1 Introduction

Malignant cerebral edema (MCE) is a severe and potentially fatal complication following large hemispheric infarction, frequently necessitating decompressive hemicraniectomy and carrying a prohibitively high mortality risk (1). Although recent randomized controlled trials (RCTs) have demonstrated the efficacy of endovascular thrombectomy (EVT) for acute ischemic stroke (AIS) (2–5), MCE remains a critical clinical challenge (6). Notably, secondary analyses of EVT trials indicate that approximately 33.9% of patients develop cerebral edema with mass effect, typically manifesting as a midline shift, despite successful reperfusion (7). This clinical paradox underscores the urgent need to optimize post-procedural management. Consequently, early identification of patients at high risk for MCE is essential to optimize the timing of intensive neuromonitoring and surgical triage. To address this, clinicians urgently require a predictive tool that is not only diagnostically accurate but also sufficiently rapid to support immediate therapeutic decision-making in high-pressure emergency environments (6, 8, 9).

Established risk factors of MCE, such as younger age, severe neurological deficits, extensive infarction volume, and unsuccessful revascularization, provide important insights but are insufficient as standalone predictors (10, 11). Consequently, there is a pressing need for rapid, objective, and integrative predictive tools. Non-contrast CT (NCCT) serves as the universal first-line modality in acute stroke, offering rapid accessibility and the potential for early quantification of brain edema (12). In immediate post-EVT NCCT scans, post-interventional cerebral hyperdensities (PCHD), defined as hyperdense parenchymal areas observed immediately after the procedure, serve as pivotal indicators of blood-brain barrier disruption (13, 14). Recent studies have validated the prognostic value of PCHD using deep learning features by specifically targeting the single axial slice exhibiting the maximum PCHD area. However, these approaches have achieved exceptional MCE risk stratification, with area under the curves (AUCs) ranging from 0.889 to 0.924 (9, 15). Nevertheless, these promising models currently lack robust external validation, thereby limiting their generalizability. Furthermore, as Wang et al. (16) indicated, relying exclusively on a single 2D slice inherently omits the spatial coherence and volumetric context present in 3D data, leading to a partial loss of prognostic information. To compensate for this inherent radiographic limitation without sacrificing the rapid processing speed vital for emergency workflows, it is imperative to enrich the predictive profile.

Emerging consensus indicates that integrating targeted 2D imaging features with comprehensive clinical data through multimodal fusion can resolve this information deficit, thereby enhancing overall prognostic accuracy (9, 17, 18). For instance, recent studies (9, 19–21) have demonstrated that integrating clinical characteristics with multi-omics imaging features significantly improves the prediction of adverse post-procedural outcomes. However, these models rely on isolated tabular variables, thereby failing to capture latent semantic interactions within patient histories. To overcome this, a natural language processing (NLP) model (BioClinicalBERT) was employed to encode synthesized clinical narratives (22). Integrating these NLP-derived semantic representations with targeted 2D deep imaging features holds the potential to generate a highly discriminative, time-efficient predictive tool for early MCE risk assessment.

Consequently, this study aims to develop a streamlined and highly efficient multimodal framework for predicting post-EVT MCE by integrating deep imaging features from the specific NCCT slice demonstrating maximum PCHD with NLP-encoded clinical narratives.

## 2 Materials and methods

### 2.1 Ethical approval of the study protocol

This retrospective multi-center cohort study was conducted across three comprehensive stroke centers in Zhejiang Province, China, between July 2016 and January 2024. The participating institutions included The Fourth Affiliated Hospital of Zhejiang University School of Medicine (Center 1), The First Hospital of Jiaxing (Center 2), and Tongde Hospital of Zhejiang Province (Center 3). All procedures adhered to the ethical guidelines of the 1964 Declaration of Helsinki and its subsequent amendments. The study protocol was reviewed and approved by the Institutional Review Boards (IRBs) of all participating hospitals (Primary Approval No. K2024139, Center 1). Given the retrospective nature of the study and the exclusive use of anonymized data, the requirement for obtaining written informed consent was waived by the respective IRBs.

### 2.2 Patient selection

Patients with AIS who underwent EVT during the study period were consecutively enrolled. The inclusion criteria were as follows: (1) age ≥18 years; (2) radiologically confirmed large vessel occlusion (LVO) in the anterior circulation; (3) underwent EVT.

Patients were excluded based on the following: (1) failed or aborted EVT procedures; (2) acquisition of the initial post-EVT NCCT exceeding 1 h; (3) lack of visible PCHD on the immediate NCCT; (4) Poor image quality due to severe motion or metallic artifacts; (5) absence of follow-up NCCT within 2–5 days for MCE assessment.

**Figure 1:**
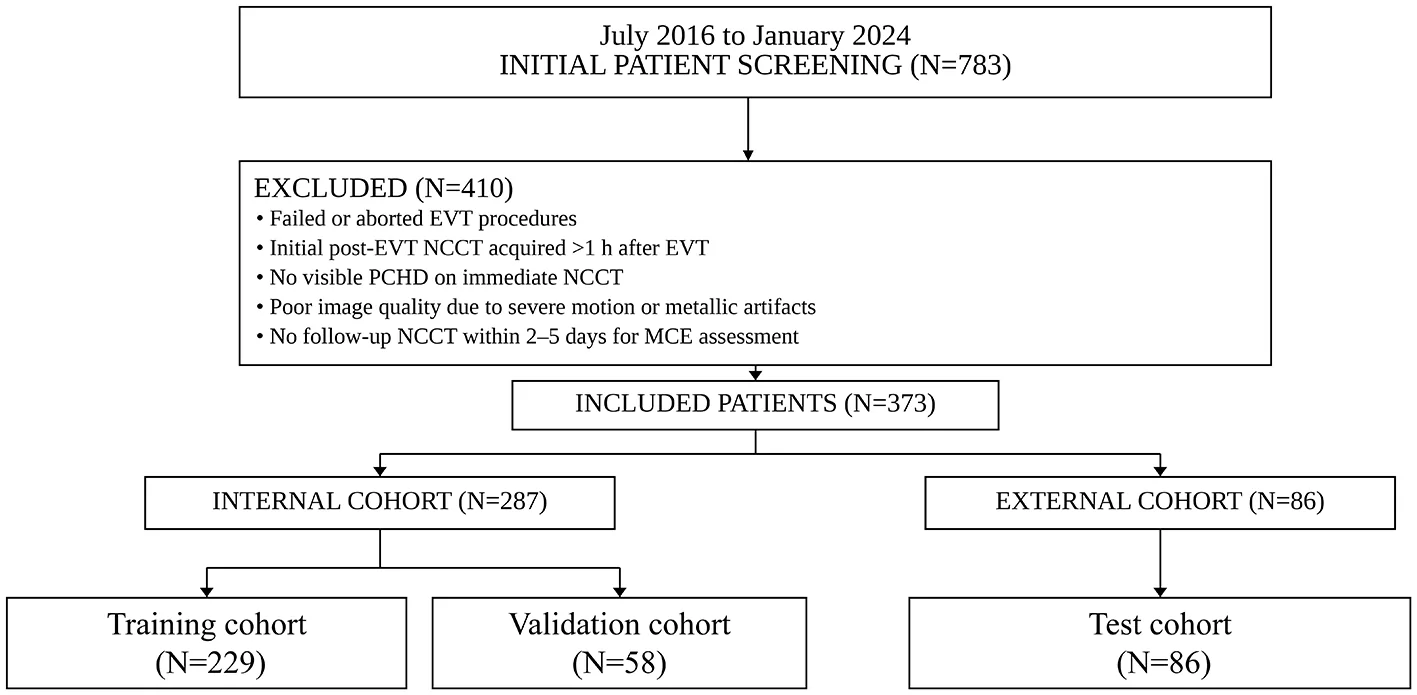
The patient selection flowchart.

The final eligible cohort was divided by institution into an internal dataset (Center 1, *n* = 287) and an external dataset (Centers 2 and 3, *n* = 86). The internal dataset was randomly partitioned into training and validation sets at a 7:3 ratio, while the external dataset was reserved exclusively for independent testing. The detailed patient selection workflow is illustrated in Figure 1.

### 2.3 Identification of PCHD and MCE

PCHD was defined as focal hyperattenuating regions within the infarcted brain parenchyma relative to adjacent tissue, excluding physiological calcifications and visible vascular structures.

The primary outcome was MCE, defined as a midline shift (MLS) of ≥5 mm on the follow-up NCCT acquired between 2 and 5 days post-procedure. MLS was measured at the level of the septum pellucidum as the maximum perpendicular distance from the anatomical midline, defined by a linear axis connecting the anterior attachment of the falx cerebri to the internal occipital protuberance.

All imaging evaluations were independently conducted by two experienced neuroradiologists who were strictly blinded to the patients' baseline clinical variables and outcomes. Discrepancies were resolved by consensus.

### 2.4 Clinical data

Comprehensive clinical data were systematically extracted from electronic medical records, procedure notes, and follow-up charts.

Baseline demographic and clinical factors included demographics (age and sex), lifestyle factors (smoking and alcohol use), and comorbidities (hypertension, hyperlipidemia, diabetes mellitus, atrial fibrillation, and coronary artery disease).

Initial neurological severity was assessed using the National Institutes of Health Stroke Scale (NIHSS). For baseline radiological assessment, the Alberta Stroke Program Early CT Score (ASPECTS) was independently evaluated on admission for NCCT by two neuroradiologists (each with >5 years of experience) strictly blinded to the patients' clinical information. Any inter-observer discrepancies were resolved through consensus.

#### 2.4.1 Treatment and procedural parameters

Treatment and procedural parameters included intravenous thrombolysis (IVT), stent deployment status, device type, number of passes, and final reperfusion status graded using the modified Thrombolysis in Cerebral Infarction (mTICI) scale. Collateral circulation status and detailed tissue-window parameters, including onset-to-puncture and onset-to-reperfusion times, were not uniformly available across the participating centers and therefore could not be incorporated. Onset-to-admission time was collected and is reported in Table 1.

**Table 1:** Baseline characteristics of patients with anterior-circulation large-vessel occlusion stroke treated with endovascular thrombectomy, stratified by malignant cerebral edema across the training, validation, and external test cohorts.

Variable	Training cohort (*n* = 229)			Validation cohort (*n* = 58)			Test cohort (*n* = 86)		
	No MCE (*n* = 176)	MCE (*n* = 53)	*P* value	No MCE (*n* = 44)	MCE (*n* = 14)	*P* value	No MCE (*n* = 64)	MCE (*n* = 22)	*P* value
Age, y	67.4 ± 14.6	65.7 ± 16.1	0.472	67.0 ± 17.0	63.6 ± 13.5	0.500	70.3 ± 14.2	74.0 ± 10.4	0.262
Male sex	108 (61.4%)	35 (66.0%)	0.650	31 (70.5%)	9 (64.3%)	0.918	40 (62.5%)	7 (31.8%)	0.025
Smoking	36 (20.5%)	12 (22.6%)	0.880	11 (25.0%)	3 (21.4%)	1.000	10 (15.6%)	2 (9.1%)	0.723
Alcohol	33 (18.8%)	13 (24.5%)	0.468	6 (13.6%)	3 (21.4%)	0.673	10 (15.6%)	2 (9.1%)	0.723
Hypertension	110 (62.5%)	27 (50.9%)	0.179	24 (54.5%)	7 (50.0%)	1.000	31 (48.4%)	12 (54.5%)	0.805
Diabetes mellitus	28 (15.9%)	8 (15.1%)	1.000	6 (13.6%)	2 (14.3%)	1.000	11 (17.2%)	1 (4.5%)	0.282
Atrial fibrillation	72 (40.9%)	23 (43.4%)	0.870	18 (40.9%)	7 (50.0%)	0.773	26 (40.6%)	10 (45.5%)	0.884
Hyperlipidemia	19 (10.8%)	4 (7.5%)	0.609	4 (9.1%)	1 (7.1%)	1.000	0 (0.0%)	1 (4.5%)	0.256
Coronary artery disease	20 (11.4%)	4 (7.5%)	0.610	6 (13.6%)	2 (14.3%)	1.000	3 (4.7%)	1 (4.5%)	1.000
Baseline NIHSS score	15.0 (11.0–19.0)	17.0 (14.0–21.0)	0.034	17.0 (13.8–20.2)	17.0 (13.2–18.0)	0.784	15.0 (12.0–18.0)	16.0 (15.0–20.8)	0.351
Baseline mRS score	5.0 (4.0–5.0)	5.0 (5.0–5.0)	0.042	5.0 (5.0–5.0)	5.0 (5.0–5.0)	0.245	5.0 (5.0–5.0)	5.0 (5.0–5.0)	0.408
Onset to admission time, min	415.0 ± 328.7	352.4 ± 230.8	0.197	287.5 ± 174.0	265.7 ± 116.6	0.664	400.1 ± 283.6	409.1 ± 278.4	0.898
ASPECTS	9.0 (8.0–10.0)	8.0 (6.0–9.0)	< 0.001	9.0 (8.0–10.0)	8.0 (7.2–10.0)	0.352	9.0 (8.8–9.0)	8.5 (8.0–9.0)	0.039
HUmax ≥ 90	35 (19.9%)	31 (58.5%)	< 0.001	11 (25.0%)	7 (50.0%)	0.153	27 (42.2%)	15 (68.2%)	0.063
TOAST classification			0.989			0.656			0.593
LAA	92 (52.3%)	27 (50.9%)		26 (59.1%)	6 (42.9%)		46 (71.9%)	14 (63.6%)	
CE	56 (31.8%)	18 (34.0%)		14 (31.8%)	6 (42.9%)		17 (26.6%)	8 (36.4%)	
Others	13 (7.4%)	4 (7.5%)		1 (2.3%)	1 (7.1%)		0 (0.0%)	0 (0.0%)	
Unknown	15 (8.5%)	4 (7.5%)		3 (6.8%)	1 (7.1%)		1 (1.6%)	0 (0.0%)	
Intravenous thrombolysis	60 (34.1%)	19 (35.8%)	0.943	17 (38.6%)	3 (21.4%)	0.338	20 (31.2%)	8 (36.4%)	0.859
Number of passes	1.0 (1.0–2.0)	2.0 (1.0–4.0)	0.012	1.0 (1.0–2.0)	2.7 ± 2.3	0.098	2.0 (1.0–2.0)	1.5 (1.0–2.8)	0.899
Aspiration catheter	70 (39.8%)	26 (49.1%)	0.297	22 (50.0%)	6 (42.9%)	0.874	17 (26.6%)	2 (9.1%)	0.136
Permanent stent implantation	35 (19.9%)	7 (13.2%)	0.369	9 (20.5%)	1 (7.1%)	0.424	0 (0.0%)	0 (0.0%)	1.000
Stent retriever type			0.257			0.245			0.682
Solitaire	96 (54.5%)	37 (69.8%)		26 (59.1%)	10 (71.4%)		4 (6.2%)	2 (9.1%)	
Trevo	24 (13.6%)	4 (7.5%)		7 (15.9%)	0 (0.0%)		14 (21.9%)	7 (31.8%)	
Solitaire + Trevo	34 (19.3%)	7 (13.2%)		9 (20.5%)	2 (14.3%)		45 (70.3%)	13 (59.1%)	
Others	22 (12.5%)	5 (9.4%)		2 (4.5%)	2 (14.3%)		1 (1.6%)	0 (0.0%)	
mTICI score ≥ 2b	166 (94.3%)	42 (79.2%)	0.002	42 (95.5%)	13 (92.9%)	1.000	57 (89.1%)	20 (90.9%)	1.000
Hemorrhagic transformation	116 (65.9%)	50 (94.3%)	< 0.001	23 (52.3%)	13 (92.9%)	0.010	43 (67.2%)	22 (100.0%)	0.001
Symptomatic ICH	61 (34.7%)	30 (56.6%)	0.007	12 (27.3%)	8 (57.1%)	0.084	26 (40.6%)	14 (63.6%)	0.105

Continuous variables are presented as mean ± SD (compared using Student's *t*-test) or median (IQR) (compared using the Mann–Whitney *U*-test). Categorical variables are presented as *n* (%) (compared using the chi-square or Fisher's exact test). *P* value in bold indicates statistical significance (*P* < 0.05). ASPECTS, Alberta Stroke Program Early CT Score; CE, cardioembolism; HUmax, maximum Hounsfield unit; ICH, intracerebral hemorrhage; IQR, interquartile range; LAA, large-artery atherosclerosis; MCE, malignant cerebral edema; mRS, modified Rankin Scale; mTICI, modified Thrombolysis in Cerebral Infarction; NIHSS, National Institutes of Health Stroke Scale; SD, standard deviation; TOAST, Trial of Org 10,172 in Acute Stroke Treatment.

Finally, missing data (< 3%) were imputed using median values for continuous variables and mode values for categorical variables.

### 2.5 CT data acquisition

All patients underwent NCCT within 1 h following the completion of EVT using multidetector CT scanners across participating centers. Standardized acquisition and reconstruction parameters were rigorously maintained across all institutions. These predominantly included an axial scanning mode, a standardized tube voltage of 120 kV (or dual-energy synthesized 120-kVp equivalent), a tube current ranging from 171 to 300 mAs, whole-head coverage, and a reconstruction slice thickness of 5.0 mm using a standard medium-smooth kernel. Comprehensive details regarding specific scanner models, vendors, and tailored dual-energy reconstruction algorithms are provided in Supplementary Material S1.

To optimize computational efficiency while preserving diagnostic relevance, the image preprocessing protocol focused on a single representative axial slice demonstrating the maximal PCHD extent. This targeted two-dimensional slice served as the exclusive input for downstream deep feature extraction. Additionally, the representative slice for each patient was independently selected by two experienced neuroradiologists, with any inter-observer discrepancies resolved through consensus.

### 2.6 Feature extraction framework

#### 2.6.1 Deep imaging feature extraction

For each CNN backbone, high-level imaging representations were extracted from the selected post-EVT NCCT slice. The raw feature vectors were first reduced to 289 latent components using principal component analysis (PCA), and seven statistical descriptors, including Sum, Mean, Median, AbsSum, L2 Norm, Mean of Squares, and Median of Squares, were then calculated to generate the final imaging signatures (DeepScores). The diagnostic label was binary, defined as MCE vs. non-MCE. ResNet101 was selected as the final imaging backbone because it achieved the highest AUC in the independent external test cohort among the four candidate architectures. To extract high-level imaging representations, the selected axial slice was standardized (window width 90 HU, window level 40 HU), resized to 224 × 224 pixels, and normalized through z-score scaling. Multiple mainstream deep learning models (ResNet-101, ResNet-50, GoogLeNet, and VGG-11) were constructed and systematically compared. The network parameters were optimized using a stochastic gradient descent (SGD) optimizer with a batch size of 32. An initial learning rate of 0.01 was applied, which was gradually decayed over 50 epochs utilizing a cosine annealing schedule.

To manage feature redundancy and prevent overfitting in downstream algorithms, the raw feature vectors were compressed into 289 intermediate latent components through principal component analysis (PCA). These were further summarized using seven statistical descriptors (Sum, Mean, Median, Sum of Absolute Values (AbsSum), L2 Norm, Mean of Squares, and Median of Squares), generating core deep-learning signatures (DeepScores).

#### 2.6.2 NLP-based clinical feature extraction

Notably, the NLP input was generated through template-based serialization of structured clinical variables rather than direct extraction from raw free-text medical records. This strategy was adopted to preserve semantic relationships among clinical variables while reducing variability associated with unstructured free-text documentation. A template-based serialization strategy was employed to transform discrete, pre-interventional clinical data (such as age, NIHSS score, and comorbidities) into coherent natural language narratives (23). BioClinicalBERT, a pre-trained NLP model, was leveraged as a fixed feature extractor with frozen pre-trained weights. The global contextual information for each patient was captured by extracting the 768-dimensional [CLS] token embedding from the model's final hidden layer.

This raw semantic representation was then projected into a more compact 512-dimensional latent space using a customized refinement block, which sequentially applied a linear layer, a Gaussian Error Linear Unit (GELU) activation function, and a dropout layer (*p* = 0.1). To align with the deep imaging feature extraction strategy, the exact same seven statistical pooling functions were computed across the 512 dimensions. Consequently, the complex clinical narratives were distilled into seven quantitative NLP signatures, priming them for multimodal integration.

### 2.7 Multimodal fusion model construction

The final predictive framework was developed leveraging a multimodal machine learning fusion strategy. To capture the comprehensive clinical profile of each patient, the integrated input feature space was constructed by concatenating three distinct components: (1) the seven ResNet-derived imaging signatures (DeepScores); (2) the seven NLP-encoded quantitative text signatures; (3) the original baseline and peri-procedural structured clinical variables. This composite feature vector was subsequently fed into a multilayer perceptron (MLP) classifier. The MLP classifier is a feed-forward neural network composed of an input layer, one or more hidden fully connected layers, and an output layer. In this study, the concatenated multimodal feature vector was used as the input. Through successive linear transformations and non-linear activation functions, the MLP learned complex interactions among ResNet-derived imaging signatures, BioClinicalBERT-derived semantic features, and structured clinical variables. The final output layer generated the predicted probability of MCE for each patient. The concatenated feature vector comprised all seven ResNet-101-derived DeepScores, seven BioClinicalBERT-derived semantic signatures, and the original structured clinical variables.

The final prediction model was based on the concatenated multimodal feature vector. SHAP analysis was subsequently applied as a *post hoc* interpretability method to quantify feature contributions and was not involved in model training or prediction. All single-modality baseline models were implemented using the same MLP architecture to ensure fair comparison across input modalities. classifier based on the concatenated multimodal feature vector. SHAP analysis was subsequently applied as a *post hoc* interpretability method to quantify feature contributions and was not involved in model training or prediction. All single-modality baseline models were implemented using the same MLP architecture to ensure fair comparison across input modalities.

**Figure 2:**
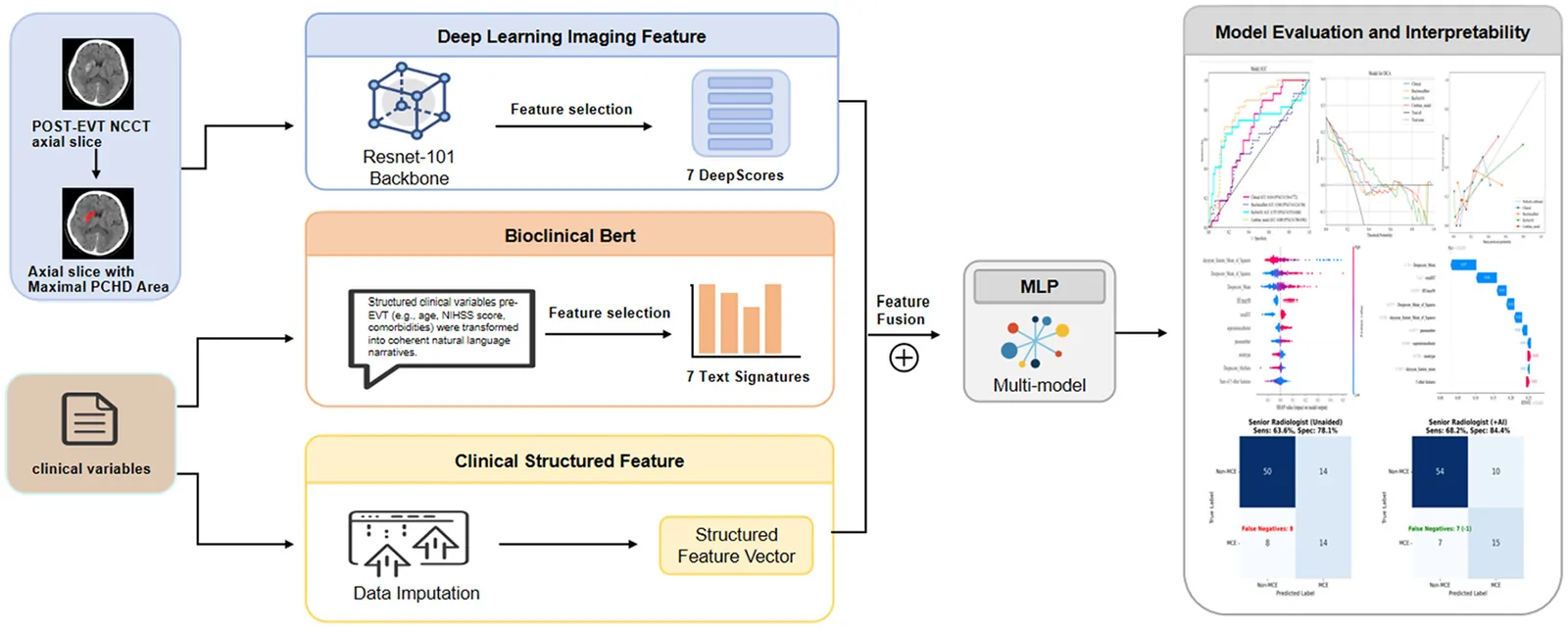
The overall framework of the proposed multimodal fusion model. CT, computed tomography; PCHD, post-thrombectomy cerebral hyperdensity; EVT, endovascular thrombectomy; NIHSS, National Institutes of Health Stroke Scale; MLP, classifier; SHAP, SHapley Additive exPlanations; MCE, malignant cerebral edema. Microsoft Corporation. “Insert icons in Microsoft 365.” License: Used in accordance with the applicable Microsoft Office/Microsoft 365 license terms. Microsoft states that its built-in icons are free to use and may be recolored, resized, and otherwise formatted. Source: Microsoft PowerPoint built-in icon library (“Insert” > “Icons”).

In summary, the computational workflow consisted of four sequential modules: (1) image preprocessing and CNN-based feature extraction, (2) clinical narrative serialization and BioClinicalBERT-based semantic embedding, (3) multimodal feature fusion and MLP classification, and (4) *post hoc* interpretability and clinical evaluation. A detailed step-by-step description of the entire computational pipeline is provided in the Supplementary Material S2. Figure 2 illustrates the proposed multimodal fusion architecture, detailing the parallel extraction of NCCT imaging features and semantic clinical narratives, followed by their concatenation for MLP-based risk classification.

To evaluate the diagnostic value of multimodal integration, the proposed fusion framework was benchmarked against three single-modality models. To isolate the effect of the input modalities and ensure a standardized comparison, all baseline models were constructed using the identical MLP architecture and data partitioning strategy. Specifically, these comprised the following: (1) a Clinical-only model, incorporating only structured baseline and peri-procedural variables; (2) a ResNet-only model, based solely on the ResNet-derived DeepScores; (3) a BERT-only model, driven exclusively by the encoded semantic text signatures.

### 2.8 Model interpretability and human-machine comparison

SHapley Additive exPlanations (SHAP) analysis was employed to visualize feature importance of the fusion model, identifying which imaging or clinical factors contributed most to the prediction of MCE. Furthermore, a Human-Machine Comparison study was conducted, as well as two neuroradiologists who independently reviewed the test cohort patients, first establishing a diagnosis based on standard clinical data, and subsequently re-evaluating the same cases with the assistance of the model's predictions. The readers' diagnostic accuracy before and after AI assistance was statistically compared to determine the model's clinical value.

### 2.9 Statistical analysis

Statistical analyses were performed in R (version 4.5.1; R Foundation for Statistical Computing, Vienna, Austria). Following normality assessment through Kolmogorov-Smirnov and Shapiro-Wilk tests, continuous data were summarized as means ± standard deviations (independent *t*-test) or medians with interquartile ranges (Mann-Whitney *U*-test), as appropriate. Categorical variables were summarized as counts and percentages, compared using the chi-square or Fisher's exact test. Computational procedures, including feature extraction and predictive modeling, were implemented in Python (version 3.11.1). Model discriminative capacity was evaluated using the AUC (with 95% CIs), accuracy, sensitivity, and specificity. To rigorously validate the incremental value of the multimodal approach, DeLong's test was utilized to assess statistically significant differences between the receiver operative characteristic (ROC) curves of the fusion model and the single-modality baselines. Statistical significance was defined as a two-tailed *P* < 0.05.

## 3 Results

### 3.1 Patient characteristics and cohort analysis

A total of 373 patients were included in the final analysis, with a mean age of 67.8 ± 14.9 years, and an overall MCE incidence of 23.9% (89/373). The dataset was divided into a training cohort (*n* = 229), a validation cohort (*n* = 58), and an external test cohort (*n* = 86). Detailed baseline characteristics stratified by MCE status are presented in Table 1. In the training cohort, patients who developed MCE exhibited significantly higher baseline NIHSS scores [median (IQR): 17.0 (14.0–21.0) vs. 15.0 (11.0–19.0), *P* = 0.034] and lower baseline ASPECTS [8.0 (6.0–9.0) vs. 9.0 (8.0–10.0), *P* < 0.001]. Procedural variables were also associated with MCE occurrence, including a higher number of thrombectomy passes (*P* = 0.012), lower rates of successful recanalization (mTICI ≥ 2b, *P* = 0.002), and the presence of high-density signs (HUmax ≥ 90, *P* < 0.001). Additionally, hemorrhagic transformation demonstrated a consistent association with MCE across all cohorts (*P* < 0.05).

### 3.2 Selection of the optimal deep learning architecture

To identify the most suitable architecture for imaging feature extraction, multiple convolutional neural networks were evaluated, including ResNet-101, ResNet-50, GoogLeNet, and VGG-11. The comparative performance of these models is summarized in the Supplementary Table S1. Among the evaluated architectures, ResNet-101 exhibited the best predictive performance in the external test cohort, achieving an AUC of 0.707 (95% CI: 0.5548–0.8599), with an accuracy of 77.9%, a sensitivity of 63.6%, and a specificity of 82.8%. In contrast, VGG-11, GoogLeNet, and ResNet-50 yielded lower AUC values of 0.501, 0.577, and 0.661, respectively.

### 3.3 Performance of the multimodal fusion model

**Figure 3:**
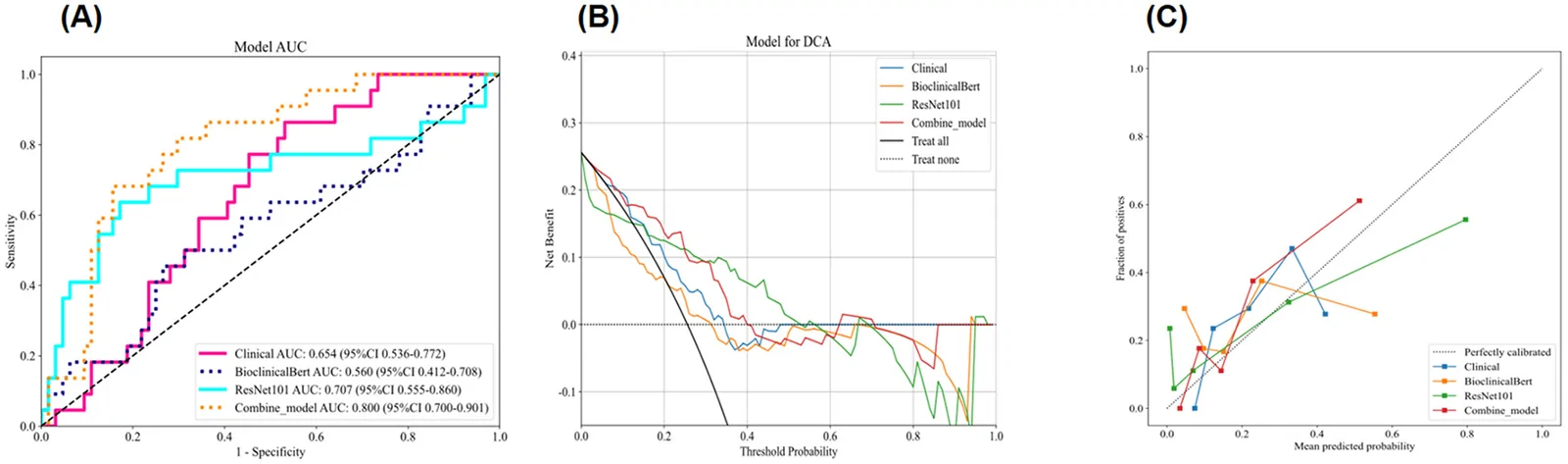
Diagnostic performance of the multimodal fusion model and single-modality models in the external test cohort. **(A)** Receiver operating characteristic (ROC) curves for the clinical-only model, BioClinicalBERT-only model, ResNet-101-only model, and combined model. The combined model achieved the highest discrimination with an AUC of 0.800 (95% CI: 0.700–0.901), compared with 0.654 for the clinical-only model, 0.560 for the BioClinicalBERT-only model, and 0.707 for the ResNet-101-only model. **(B)** Decision curve analysis (DCA) of the four models across threshold probabilities. **(C)** Calibration curves showing the agreement between predicted probabilities and observed outcomes.

The predictive performance of the multimodal fusion model and the three single-modality models is summarized in Table 2. To further demonstrate the incremental value of multimodal integration, a multi-dimensional performance comparison is visualized in Figure S1. While single-modality models exhibit diagnostic trade-offs, the fusion model maintains an expansive and balanced profile across AUC, accuracy, sensitivity, and specificity, particularly in the independent external cohort. In the independent external test cohort, the multimodal fusion model achieved an AUC of 0.800 (95% CI: 0.6996–0.9013), with an accuracy of 80.2%, sensitivity of 68.2%, specificity of 84.4%, outperforming the clinical-only model (AUC = 0.654), the ResNet-only model (AUC = 0.707), and the BERT-only model (AUC = 0.560). ROC curves for external datasets are presented in Figure 3.

**Table 2:** Diagnostic performance of the multimodal fusion model and the three single-modality models for predicting malignant cerebral edema in the training and external test cohorts.

Signature	Cohort	Accuracy	AUC	95% CI	Sensitivity	Specificity	PPV	NPV	Precision	Recall	F1	Threshold
Clinical	Train	0.751	0.804	0.7416–0.8664	0.755	0.750	0.476	0.910	0.476	0.755	0.584	0.243
	Test	0.570	0.654	0.5358–0.7724	0.864	0.469	0.358	0.909	0.358	0.864	0.507	0.157
BioClinicalBERT	Train	0.790	0.867	0.8119–0.9212	0.811	0.784	0.531	0.932	0.531	0.811	0.642	0.229
	Test	0.663	0.560	0.4123–0.7084	0.455	0.734	0.370	0.797	0.370	0.455	0.408	0.243
ResNet101	Train	0.974	0.993	0.9839–1.0000	0.943	0.983	0.943	0.983	0.943	0.943	0.943	0.412
	Test	0.779	0.707	0.5548–0.8599	0.636	0.828	0.560	0.869	0.560	0.636	0.596	0.354
Combined model	Train	0.847	0.940	0.9081–0.9727	0.906	0.830	0.615	0.967	0.615	0.906	0.733	0.240
	Test	0.802	0.800	0.6996–0.9013	0.682	0.844	0.600	0.885	0.600	0.682	0.638	0.240

The clinical model was based on structured baseline clinical variables; the BioClinicalBERT model was based on semantic embeddings derived from unstructured clinical notes; the ResNet-101 model was based on deep imaging features extracted from post-thrombectomy non-contrast CT; and the Combined model integrated all three feature sources through late fusion. AUC, area under the receiver operating characteristic curve; BioClinicalBERT, Bidirectional Encoder Representations from Transformers pretrained on clinical notes; CI, confidence interval; CNN, convolutional neural network; F1, F1 score; NPV, negative predictive value; PPV, positive predictive value.

### 3.4 Model interpretability and feature contributions

**Figure 4:**
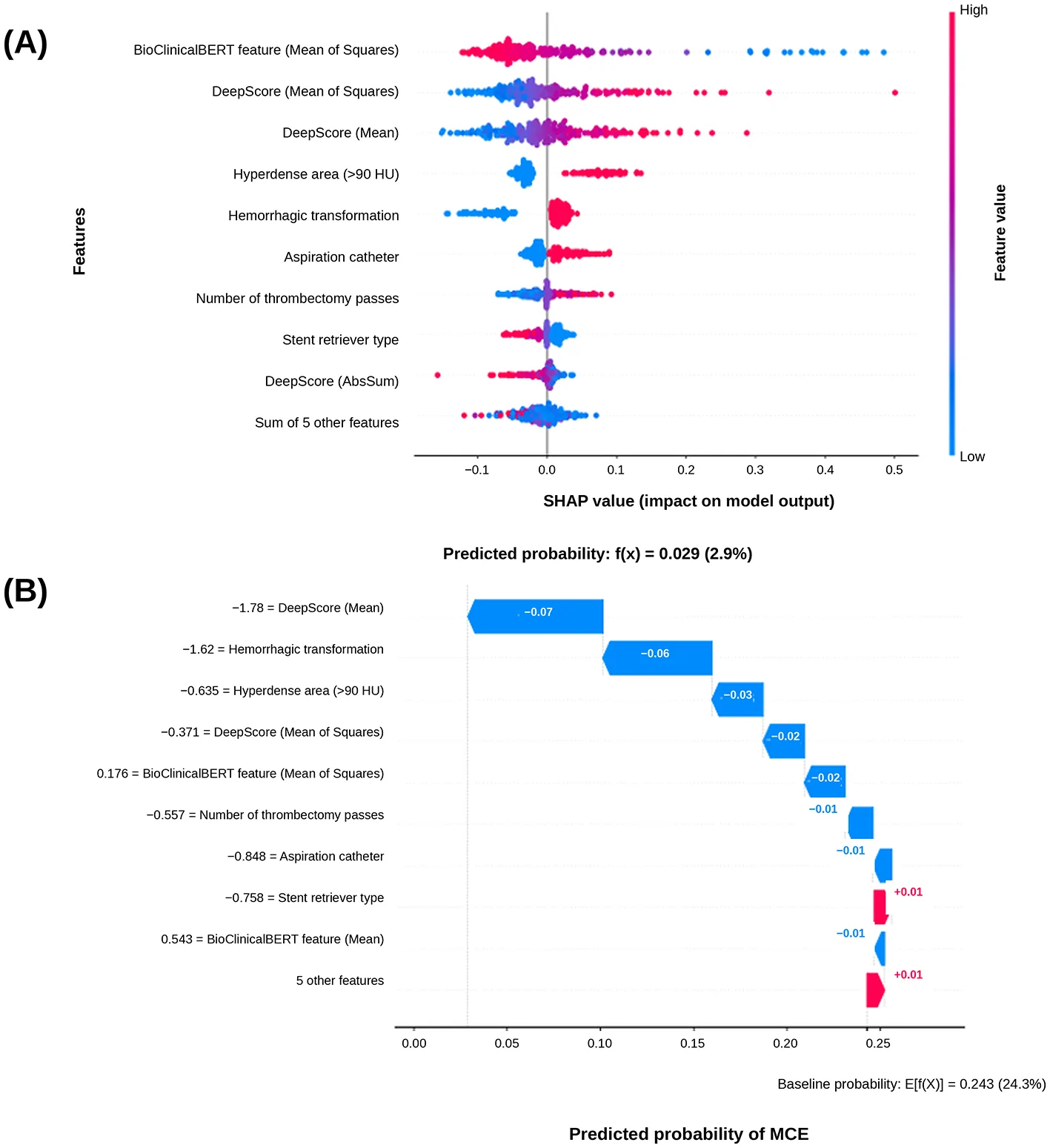
Interpretability analysis of the multimodal fusion model using SHapley Additive exPlanations (SHAP). **(A)** SHAP summary plot showing the global importance and directional effects of the most influential features. The NLP-derived semantic feature ranked first, followed by DeepScore-related features and clinically interpretable variables such as hyperdense areas (>90 HU), hemorrhagic transformation, and the number of thrombectomy passes. **(B)** Representative SHAP waterfall plot for a patient predicted to be at low risk of malignant cerebral edema (predicted probability = 0.029), illustrating how individual features contributed to reducing the final risk estimate.

The contribution of individual features to the multimodal model was evaluated using SHAP. The summary plot (Figure 4A) demonstrated that the NLP-derived semantic feature was the most influential predictor. Lower values (blue dots) of this feature were associated with higher SHAP values, indicating increased risk of MCE. The DeepScores (specifically, Mean and Variance) ranked second and third, demonstrating a positive correlation where higher values (red dots) drove higher risk probabilities. In the SHAP-based feature-importance analysis of the fusion model, several ResNet-101-derived DeepScores, including Mean of Squares, Mean, and AbsSum, ranked among the top nine contributing predictors, further confirming the meaningful contribution of imaging descriptors to the multimodal prediction. Furthermore, clinically interpretable variables demonstrated strong positive directionality; notably, the presence of hyperdense areas (>90 HU), hemorrhagic transformation, and a higher number of stent retriever passes were all associated with increased MCE risk.

At the individual level, a representative SHAP waterfall plot (Figure 4B) illustrated the contribution of each feature to prediction. In this case, a low DeepScore value (SHAP = −0.07) and absence of hemorrhagic transformation (SHAP = −0.06) contributed negatively to the prediction, resulting in a low estimated probability of MCE.

### 3.5 Human-machine diagnostic comparison

**Figure 5:**
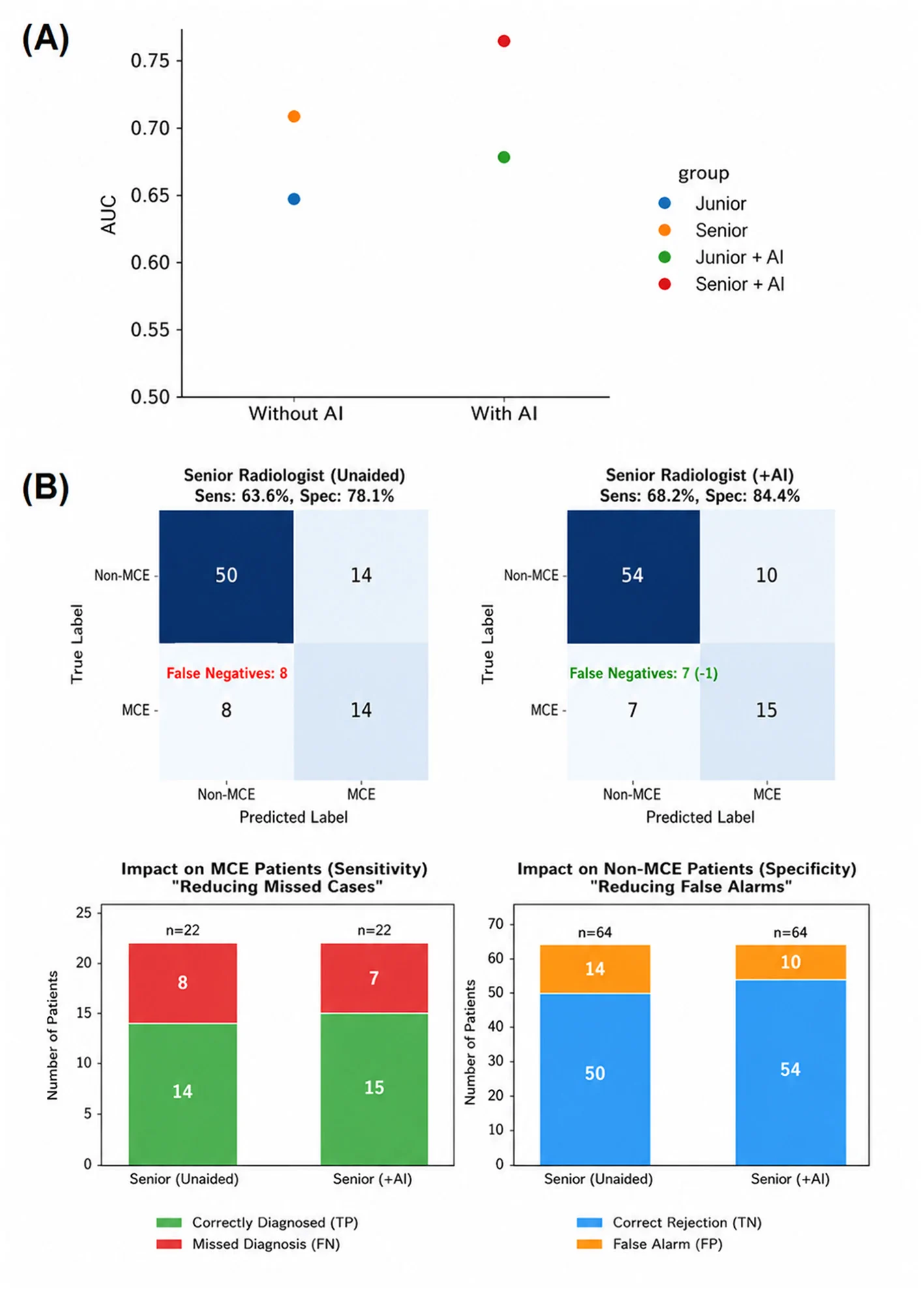
**(A)** ROC curves comparing the diagnostic performance of senior neuroradiologists with and without AI assistance. **(B)** Confusion matrices illustrating the clinical impact of AI assistance. The model acted as a robust “second reader,” helping senior experts reduce False Negatives (8 vs. 7) while simultaneously decreasing False Positives (14 vs. 10), demonstrating a “double benefit” in sensitivity and specificity.

The diagnostic performance of neuroradiologists was evaluated before and after integrating AI assistance (Figure 5A, Table 3) using a subset of the test cohort. Without assistance, senior neuroradiologists achieved an AUC of 0.709 (95% CI: 0.594–0.824) with an accuracy of 74.4%, while junior neuroradiologists achieved an AUC of 0.647 (95% CI: 0.528–0.766) with an accuracy of 67.4%. With AI assistance, the junior group's AUC improved to 0.678 (95% CI: 0.561–0.796), while their overall accuracy and specificity increased to 72.1 and 76.6%, respectively. For senior readers, AI augmentation significantly increased the AUC to 0.763 (95% CI: 0.654–0.872) and overall accuracy to 80.2%. Confusion matrix analysis (Figure 5B) revealed a reduction in both missed diagnoses (false negatives decreased from 8 to 7) and false-positive cases, which dropped by 28.6% (from 14 to 10). Consequently, senior readers exhibited concurrent enhancements in diagnostic sensitivity (from 63.6 to 68.2%), specificity (from 78.1 to 84.4%), and positive predictive value (from 50.0 to 60.0%).

**Table 3:** Diagnostic performance of neuroradiologists with and without artificial intelligence assistance compared with the multimodal AI model, evaluated on a subset of the external test cohort.

Reader	Accuracy	AUC	95% CI	Sensitivity	Specificity	PPV	NPV	Precision	Recall	F1	Threshold
AI model	0.802	0.800	0.6996–0.9013	0.682	0.844	0.600	0.885	0.600	0.682	0.638	0.24
Junior reader	0.674	0.647	0.5277–0.7663	0.591	0.703	0.406	0.833	0.406	0.591	0.481	1.00
Junior reader + AI	0.721	0.678	0.5608–0.7957	0.591	0.766	0.464	0.845	0.464	0.591	0.520	1.00
Senior reader	0.744	0.709	0.5940–0.8236	0.636	0.781	0.500	0.862	0.500	0.636	0.560	1.00
Senior reader + AI	0.802	0.763	0.6536–0.8720	0.682	0.844	0.600	0.885	0.600	0.682	0.638	1.00

The AI model corresponds to the multimodal fusion model combining ResNet-101 imaging features and BioClinicalBERT clinical text embeddings. Junior and senior readers were asked to independently predict malignant cerebral edema first without and then with access to the AI model's probability output. AI, artificial intelligence; AUC, area under the receiver operating characteristic curve; CI, confidence interval; F1, F1 score; NPV, negative predictive value; PPV, positive predictive value.

## 4 Discussion

In this multi-center study, a multimodal model to predict MCE after EVT was developed and validated. By integrating deep imaging features from a single representative CT slice with NLP-derived clinical text signatures, the combined model outperformed single-modality models. Furthermore, the model maintained robust predictive performance in an independent external cohort (AUC of 0.800, 95% CI: 0.6996–0.9013). The preservation of this diagnostic accuracy across diverse centers underscores the reliability of integrating multimodal data to predict MCE.

The SHAP analysis identified the NLP-derived feature as the predominant predictor for MCE. Specifically, the top-ranking NLP feature, BioClinicalBERT_feature_Mean_of_Squares, captured the overall semantic severity of the admission narratives. Alongside these embeddings, explicit clinical variables—including total hemorrhagic transformation, HUmax90, aspiration catheter type, and the number of thrombectomy passes—were clearly identifiable as key drivers of the prediction. This finding corroborates the work of Ji et al. (23), which demonstrated that serializing clinical variables into natural language narratives enables transformer-based architectures to outperform traditional image-only models. By projecting discrete variables, such as NIHSS and age, into the continuous semantic space of BioClinicalBERT, the combined model effectively captures global clinical context and complex variable interactions that remain latent in independent tabular datasets. Recent evidence has confirmed that NLP models demonstrate superior capacity in extracting stroke-specific phenotypes from clinical documentation (24). The results extend these findings specifically to the prediction of MCE, suggesting that the clinical narratives decoded by these models provide a more holistic representation of the patient's systemic state than radiographic data alone. This is consistent with the observations of Song et al. (25), who reported that multimodal workflows integrating unstructured clinical notes with imaging reports significantly outperform conventional approaches in identifying complex stroke phenotypes, such as large vessel occlusions.

Regarding radiological analysis, the strategy focused on extracting deep features from a single representative axial slice exhibiting maximal PCHD. This targeted approach balances computational efficiency with diagnostic relevance, making it particularly suitable for a time-sensitive clinical environment. However, the empirical comparison of multiple convolutional neural networks confirmed ResNet-101 as the optimal foundational architecture. This architecture enabled the extraction of sub-visual textural patterns that correlated strongly with MCE risk, reinforcing the previous findings (9) that deep learning-derived radiomics features from post-EVT CT outperform conventional visual scoring. Furthermore, the approach employed in this study ensures high diagnostic accuracy as well as the rapid throughput required in emergency clinical settings (26).

The human-machine comparison highlighted the model's practical value in a clinical setting. Unaided, senior neuroradiologists achieved a baseline sensitivity of 63.6% and a specificity of 78.1%. With AI assistance, their overall diagnostic performance significantly improved, evidenced by an AUC increase from 0.709 to 0.763. Confusion matrix analysis confirmed that this improvement was not a diagnostic trade-off. Furthermore, the AI achieved a simultaneous improvement in both specificity (to 84.4%) and sensitivity (to 68.2%) by reducing false positive alerts and capturing subtle MCE initially skipped by human readers. This balanced diagnostic performance directly enhances the clinical management of MCE by expanding Therapeutic time window. Consequently, clinicians are empowered to proactively initiate targeted interventions, ranging from intensive neuromonitoring to hyperosmolar therapy, well before the onset of mass effect and irreversible secondary brain injury (27, 28). Additionally, patient-specific SHAP waterfall plots (Figure 3B) establish a transparent decision pathway. Visualizing the specific features driving each prediction allows clinicians to objectively validate the AI's risk stratification before clinical integration.

Certain limitations warrant acknowledgment. First, despite the multi-center design, the study is retrospective, and selection bias regarding patients who underwent follow-up imaging is unavoidable. Second, the current methodology necessitates manual or semi-automated identification of the representative CT slice, which currently precludes a fully automated clinical workflow. Future research should address this by integrating an upstream object detection module to achieve end-to-end automation. Third, the diagnostic yield of the BioClinicalBERT model is highly contingent upon the granularity of the admission notes. Consequently, sparse documentation or overly rigid templates within certain electronic health record (EHR) systems may attenuate its predictive utility. Furthermore, although the model achieved encouraging performance in the independent external cohort, the external test sample size remained relatively modest (*n* = 86). This may limit the precision of performance estimates and preclude definitive conclusions regarding generalizability across broader healthcare systems, imaging protocols, and patient populations. Therefore, prospective validation in larger, geographically diverse, and more heterogeneous multicenter cohorts is warranted before routine clinical deployment. The use of a single representative slice was intentionally designed to reduce computational burden and facilitate rapid clinical implementation in emergency post-EVT workflows. Nevertheless, this two-dimensional strategy may omit volumetric information, including lesion volume, spatial continuity, and craniocaudal extension of PCHD. Future studies should investigate whether multi-slice or fully three-dimensional approaches, such as 3D CNNs or volumetric radiomics, can further improve predictive performance while maintaining clinical feasibility. Additionally, several important mechanistic determinants of malignant edema, such as collateral circulation status, onset-to-reperfusion time, and perfusion-derived tissue-window parameters, were not uniformly available across centers. Their absence may have limited the biological comprehensiveness of the model and should be addressed in future prospective studies. Beyond deep neural networks, other data-driven predictive paradigms, such as Gaussian Process Regression, have also been applied to complex prediction tasks and may provide useful perspectives on model generalization and uncertainty quantification (29). Although developed in a different application domain, such approaches highlight the broader importance of reliable and uncertainty-aware predictive frameworks.

In conclusion, integrating targeted NCCT imaging features with NLP-encoded clinical representations yielded a promising multimodal tool for early MCE prediction after EVT. The model showed encouraging external validation performance and improved human diagnostic performance, although prospective validation in larger and more heterogeneous cohorts remains necessary before routine clinical deployment.

## Data Availability

The datasets analyzed during the current study are not publicly available due to institutional data protection policies, patient privacy regulations, and restrictions imposed by the ethics approvals of the participating institutions. Aggregated data or selected feature representations may be available from the corresponding author upon reasonable request, subject to institutional ethical review and the execution of an appropriate data transfer agreement. Requests to access the data should be directed to Jian Ding, dingjian324@126.com.
