# Correction: Malignant cerebral edema after endovascular thrombectomy: a multimodal prediction model based on post-thrombectomy cerebral hyperdensity and natural language processing

**DOI:** 10.3389/fmed.2026.1976286

**Published:** 2026-09-02

**Authors:** Guolan Song, Jiahong Fu, Yuhan Chen, Yujie Shen, Jiayi Hong, Feifan Liu, Shiying Gai, Huan Liu, Dekuai Tong, Song Cheng, Jun Han, Jingjing Fu, Jian Ding

**Affiliations:** 1 Department of Radiology, The Fourth Affiliated Hospital of School of Medicine, International School of Medicine, International Institutes of Medicine, Zhejiang University, Yiwu, Zhejiang, China; 2 Department of Neurology, The Fourth Affiliated Hospital of School of Medicine, International School of Medicine, International Institutes of Medicine, Zhejiang University, Yiwu, Zhejiang, China; 3 Department of Neurointervention, The Fourth Affiliated Hospital of School of Medicine, International School of Medicine, International Institutes of Medicine, Zhejiang University, Yiwu, Zhejiang, China; 4 Department of Radiology, The First Hospital of Jiaxing, The Affiliated Hospital of Jiaxing University, Jiaxing, Zhejiang, China; 5 Department of Radiology, Tongde Hospital of Zhejiang Province, Hangzhou, Zhejiang, China

**Keywords:** deep learning, endovascular thrombectomy, malignant cerebral edema, multimodal fusion, natural language processing

Affiliation 5 was erroneously assigned to author Jian Ding. Author Jian Ding should be affiliated with Affiliation 4.

A correction has been made to a spelling error in Affiliation 4:

“The Affiliated Hospital of Jiaxing University”

The original version of this article has been updated.

