# Supplementary material for: Malignant cerebral edema after endovascular thrombectomy: a multimodal prediction model based on post-thrombectomy cerebral hyperdensity and natural language processing
Source: Front Med (Lausanne). 2026 Aug 5;13:1857047. doi: 10.3389/fmed.2026.1857047 (PMC13485725; doi:10.3389/fmed.2026.1857047)
Supplement: Supplementary file 1 [file Table_1.docx]

Supplemental File for Review

Supplemental Table 1. Detailed list of CT^1^ scanners used in participating centers

| Center | Manufacturer | Model | Row Number |
| --- | --- | --- | --- |
| Centre 1 | Siemens Healthineers | SOMATOM Definition AS | 64 |
|  | GE HealthCare | Optima CT620 | 62 |
| Centre 2 | United Imaging Healthcare | uCT 880 | 80 |
|  | Siemens Healthineers | SOMATOM Definition AS | 64 |
|  | United Imaging Healthcare | uCT 520 | 16 |
|  | Philips Healthcare | Ingenuity Flex | 16 |
| Centre 3 | Siemens Healthineers | SOMATOM Definition AS | 64 |
|  | GE HealthCare | Optima CT680 | 64 |

^1^CT = computed tomography.
