# Supplementary material for: Malignant cerebral edema after endovascular thrombectomy: a multimodal prediction model based on post-thrombectomy cerebral hyperdensity and natural language processing
Source: Front Med (Lausanne). 2026 Aug 5;13:1857047. doi: 10.3389/fmed.2026.1857047 (PMC13485725; doi:10.3389/fmed.2026.1857047)
Supplement: Supplementary file 2 [file Table_2.docx]

**Supplementary Material S2. Detailed Computational Workflow for Software Operation Processing**

**S2.1 Overview of the Four-Module Computational Framework**

The complete computational pipeline for the proposed multimodal malignant cerebral edema (MCE) prediction model was organized into four sequential modules, each representing a distinct analytical stage:

Module 1 — Image preprocessing and CNN-based feature extraction. Post-thrombectomy non-contrast CT (NCCT) slices were standardized, normalized, and processed through a ResNet-101 convolutional neural network to derive quantitative imaging signatures, termed DeepScores.

Module 2 — Clinical narrative serialization and BioClinicalBERT-based semantic embedding. Structured clinical variables recorded at admission were transformed into standardized narrative text via a template-based serialization strategy. These narratives were subsequently encoded into fixed-length semantic embeddings using a pretrained BioClinicalBERT model.

Module 3 — Multimodal feature fusion and MLP classification. Imaging-derived DeepScores, NLP-derived semantic signatures, and the original structured clinical variables were concatenated into a unified feature vector and entered into a multilayer perceptron classifier for binary prediction of MCE (defined as midline shift greater than or equal to 5 mm).

Module 4 — Post hoc interpretability and clinical evaluation. SHapley Additive exPlanations (SHAP) were applied as a post hoc method to quantify the contribution of individual features to model predictions. Model outputs were subsequently benchmarked against independent assessments by two neuroradiologists.

Figure 2 in the main text provides a schematic overview of the four-module architecture. The detailed step-by-step workflow is provided below.

**S2.2 Step-by-Step Computational Workflow**

**Step 1. Data Preparation and Import**

**Purpose:** Consolidate demographic, clinical, procedural, and imaging data from multiple centers into a structured analytical dataset suitable for model development and validation.

**Input:** Comma-separated value (CSV) files containing structured clinical variables exported from the electronic health record system; DICOM-formatted post-thrombectomy NCCT images retrieved from the institutional picture archiving and communication system (PACS); peri-procedural documentation and radiological reports.

**Main operation:** Clinical variables were imported and harmonized across contributing centers using a predefined data dictionary that standardized variable names, units, and coding schemes. DICOM images were matched to corresponding clinical records via unique patient identifiers and examination timestamps. Missing values were systematically documented. The complete dataset was partitioned into training, validation, and independent external test cohorts prior to any feature processing, with stratification by MCE outcome and data source to preserve distributional balance across subsets.

**Output:** A cleaned, matched multimodal dataset indexed by patient identifier, together with saved data partition indices defining the training, validation, and external test cohorts.

**Quality control / reproducibility consideration:** All data import and harmonization scripts were version-controlled. The random seed used for cohort partitioning was recorded to ensure reproducibility of the exact cohort composition.

**Step 2. Image Preprocessing**

**Purpose:** Standardize post-thrombectomy NCCT slices to ensure consistent spatial dimensions and intensity distributions across scanners and study centers, thereby reducing technical variability prior to CNN-based feature extraction.

**Input:** A single axial NCCT slice demonstrating the maximal craniocaudal extent of post-thrombectomy cerebral hyperdensity (PCHD), independently selected by two neuroradiologists for each patient.

**Main operation:** DICOM pixel data were extracted and converted to Hounsfield Unit (HU) values using the rescale slope and intercept stored in the DICOM header. The image was windowed to a width of 90 HU and a level of 40 HU to optimize soft-tissue contrast in the hyperdensity range. The windowed image was resized to a fixed spatial resolution using bilinear interpolation and subsequently normalized via z-score scaling computed on a per-slice basis.

**Output:** A standardized single-channel grayscale image with consistent dimensions and z-score-normalized pixel intensities, ready for input to the CNN feature extraction module.

**Quality control / reproducibility consideration:** Slice selection was performed independently by two neuroradiologists; disagreements were resolved by consensus. The identical windowing, resizing, and normalization parameters were applied to images from all cohorts without modification.

**Step 3. CNN-Based Imaging Feature Extraction**

**Purpose:** Extract high-level deep imaging representations from preprocessed NCCT slices using a convolutional neural network and derive compact, quantitative imaging signatures (DeepScores) for downstream multimodal fusion.

**Input:** Standardized single-channel images produced by Step 2.

**Main operation:** Multiple mainstream CNN architectures, including ResNet-101, ResNet-50, GoogLeNet, and VGG-11, were evaluated as candidate imaging feature extractors. For each architecture, network weights were initialized from a large-scale natural-image dataset and subsequently fine-tuned on the training cohort. High-level feature representations were extracted from the penultimate fully connected layer. The resulting high-dimensional feature vectors were compressed via principal component analysis to a reduced set of latent components, retaining the majority of cumulative variance. Seven statistical descriptors were then calculated from these latent components to generate a compact set of quantitative imaging signatures, termed DeepScores (Sum, Mean, Median, AbsSum, L2 Norm, Mean of Squares, and Median of Squares). Among the candidate architectures, ResNet-101 was selected as the final imaging backbone on the basis of its external test cohort performance. All seven ResNet-101-derived DeepScores were incorporated into the final fusion model.

**Output:** Seven DeepScore values per patient, representing a compressed statistical summary of the deep imaging features extracted by the ResNet-101 backbone.

**Quality control / reproducibility consideration:** The PCA transformation was computed exclusively on the training cohort and applied without refitting to the validation and external test cohorts, preventing data leakage. The same set of seven statistical descriptors was applied uniformly across all candidate CNN architectures to ensure a standardized comparison.

**Step 4. Clinical Narrative Preparation**

**Purpose:** Transform structured clinical variables into standardized narrative text suitable for processing by a pretrained biomedical language model, while reducing the variability that would be introduced by raw free-text clinical documentation.

**Input:** Structured clinical variables collected at admission, encompassing demographics, vascular risk factors, lifestyle factors, baseline neurological deficit severity (NIHSS), baseline ASPECTS, intravenous thrombolysis status, endovascular treatment variables (stent retriever type, number of passes, final mTICI grade), and post-thrombectomy imaging markers (maximum Hounsfield Unit value, hemorrhagic transformation). The complete variable list is provided in Table 1 of the main text.

**Main operation:** A template-based serialization strategy was adopted, in which each structured variable was embedded into a fixed-sentence template to produce a coherent clinical narrative. Categorical variables were expanded into natural-language descriptive phrases. Continuous variables were expressed in clinically conventional units and formats. The template structure was applied identically to all patients across all cohorts, ensuring lexical and syntactic consistency of the input text.

**Output:** One template-serialized clinical narrative per patient, consisting of a standardized English paragraph of consistent structure and vocabulary.

**Quality control / reproducibility consideration:** Template consistency was verified programmatically prior to model ingestion. All narratives were generated using the identical template, precluding cohort-specific or patient-specific variation in text structure.

**Step 5. BioClinicalBERT-Based Semantic Embedding**

**Purpose:** Encode template-serialized clinical narratives into fixed-length contextual embeddings that capture global semantic relationships among clinical variables, producing quantitative NLP-derived signatures for multimodal integration.

**Input:** Template-serialized clinical narratives produced by Step 4.

**Main operation:** A pretrained BioClinicalBERT model was loaded from a public model repository(https://huggingface.co/emilyalsentzer/Bio_ClinicalBERT). Each clinical narrative was tokenized using the corresponding BioClinicalBERT tokenizer and processed through the transformer encoder. The embedding corresponding to the special classification token was extracted from the final hidden layer as a document-level semantic representation. As with the imaging pipeline, the resulting embedding was compressed via principal component analysis, and seven statistical descriptors were computed to produce a compact set of quantitative NLP signatures that paralleled the structure of the imaging-derived DeepScores.

**Output:** Seven NLP-derived semantic signatures per patient, quantitatively summarizing the clinical narrative content at the document level.

**Quality control / reproducibility consideration:** BioClinicalBERT was used as a frozen feature extractor without fine-tuning on the study data, preserving the model's generalizability and avoiding overfitting to the study cohort. The PCA-based compression and descriptor calculation mirrored the imaging pipeline to maintain methodological symmetry between modalities.

**Step 6. Feature Normalization and Harmonization**

**Purpose:** Normalize all feature sources to a common numerical scale prior to fusion, ensuring that no single modality or variable dominates the MLP training process solely because of differences in native numerical range.

**Input:** Seven ResNet-101-derived imaging DeepScores (Step 3); seven BioClinicalBERT-derived NLP semantic signatures (Step 5); and the original set of structured baseline and peri-procedural clinical variables.

**Main operation:** Continuous features were standardized to zero mean and unit variance using normalization parameters computed exclusively on the training cohort. Categorical variables were encoded using a one-hot representation. Features with a high proportion of missing values across the pooled cohort were identified and excluded from the model.

**Output:** A fully numeric, normalized feature matrix of dimensions patients-by-features, where each column has been appropriately scaled or encoded for downstream modeling.

**Quality control / reproducibility consideration:** Normalization parameters (means and standard deviations for continuous features) were saved after fitting on the training cohort and applied without recomputation to the validation and external test cohorts.

**Step 7. Multimodal Feature Fusion**

**Purpose:** Integrate the three distinct feature sources (imaging DeepScores, NLP semantic signatures, and structured clinical variables) into a single unified representation for classification.

**Input:** Normalized features from Step 6: seven imaging DeepScores, seven NLP signatures, and encoded structured clinical variables.

**Main operation:** The three feature groups were concatenated horizontally — a late-fusion strategy — to form a single composite feature vector per patient. No feature-level weighting, gating, or attention mechanism was applied during concatenation, preserving the independent contribution of each data modality to the final prediction.

**Output:** A single concatenated multimodal feature vector per patient, containing all imaging, semantic, and clinical features.

**Quality control / reproducibility consideration:** The concatenation order was fixed across all experiments and recorded in the analysis pipeline. Single-modality baseline models were constructed by subsetting the fused vector to the relevant feature group, ensuring that any performance comparison reflected differences in input data rather than differences in model architecture.

**Step 8. MLP Classifier Construction and Training**

**Purpose:** Train a multilayer perceptron classifier on the concatenated multimodal feature vector to perform binary prediction of MCE after endovascular thrombectomy.

**Input:** Concatenated multimodal feature vectors from Step 7 and binary outcome labels (MCE versus non-MCE).

**Main operation:** A multilayer perceptron architecture was constructed, consisting of an input layer matching the dimensionality of the fused feature vector, one or more hidden layers with rectified linear unit activation, and a single-output neuron with sigmoid activation for binary probability estimation. Dropout regularization was applied during training. Network weights were optimized using a standard gradient-based optimizer with a binary cross-entropy loss function. Training was performed on the training cohort, with the validation cohort reserved for early-stopping decisions and hyperparameter selection. To enable fair comparison, all single-modality baseline models (Clinical-only, ResNet-only, BERT-only) were implemented using the identical MLP architecture and training procedure.

**Output:** A trained MLP classifier capable of producing a predicted probability of MCE for each patient.

**Quality control / reproducibility consideration:** The random seed governing weight initialization was fixed and recorded. Training data were used exclusively for weight optimization; the validation cohort was used only for early-stopping decisions, and the external test cohort remained completely unseen throughout model development and threshold determination.

**Step 9. Model Evaluation**

**Purpose:** Assess the discriminative performance, calibration quality, and clinical utility of the multimodal fusion model and the three single-modality baseline models.

**Input:** Trained MLP classifiers (fusion model and single-modality baselines); validation and external test cohort data with known MCE outcomes.

**Main operation:** The optimal binary classification threshold was determined by maximizing the Youden index on the training cohort and was applied without modification to the validation and external test cohorts. Model discrimination was assessed using the area under the receiver operating characteristic curve with 95 percent confidence intervals. Sensitivity, specificity, positive predictive value, negative predictive value, accuracy, and F1 score were computed at the Youden-derived threshold. Pairwise comparisons of ROC curves between the fusion model and each baseline were performed using the DeLong test. Calibration was evaluated both graphically and quantitatively. Decision curve analysis was employed to estimate net clinical benefit across a clinically relevant range of threshold probabilities.

**Output:** A comprehensive set of performance metrics for all models, including AUC values, confusion-matrix-derived statistics, calibration assessments, and decision curve analysis results (Tables 2 and 3, Figure 3 in the main text).

**Quality control / reproducibility consideration:** The classification threshold was locked after determination on the training set and never updated using external test data. All evaluation metrics were computed through a single, version-controlled analysis script to prevent manual calculation discrepancies.

**Step 10. Post Hoc Interpretability and Clinical Evaluation**

**Purpose:** Quantify the contribution of individual features to model predictions using SHAP, and benchmark the diagnostic value of AI assistance against independent assessments by neuroradiologists.

**Input:** The trained multimodal fusion model; external test cohort data; independent MCE assessments by two neuroradiologists (one junior, one senior).

**Main operation:** SHAP values were computed for all features in the external test cohort as a post hoc interpretability analysis; SHAP was not involved in model training or threshold determination. Global feature importance was visualized using a summary plot, and individual patient predictions were explained using waterfall plots. For the human-machine comparison, each neuroradiologist independently reviewed a subset of external test cohort patients, first based on standard clinical data alone and subsequently with access to the model predicted probability. Diagnostic performance metrics were computed for each reader under both conditions and compared against the standalone AI model.

**Output:** SHAP summary and waterfall plots (Figure 4, main text); human-machine diagnostic comparison metrics and confusion matrices (Figure 5 and Table 3, main text).

**Quality control / reproducibility consideration:** SHAP values were computed exclusively on the external test cohort. Human readers were blinded to true outcome labels and to each other's assessments during the independent review phase. The AI model probability was presented identically to both readers during the assisted phase.

**S2.3 Summary Table of Software Operations**

| **Step** | **Module** | **Task** | **Key Operational Description** |
| --- | --- | --- | --- |
| 1 | — | Data preparation and import | Multi-center data harmonization; DICOM-to-clinical record matching; cohort partitioning with outcome and center stratification |
| 2 | Module 1 | Image preprocessing | NCCT slice selection by dual-reader consensus; HU windowing (90/40); spatial resizing; z-score intensity normalization |
| 3 | Module 1 | CNN imaging feature extraction | Multi-architecture comparison (ResNet-101, ResNet-50, GoogLeNet, VGG-11); PCA dimensionality reduction; seven statistical descriptors as DeepScores |
| 4 | Module 2 | Clinical narrative preparation | Template-based serialization of structured clinical variables; uniform narrative structure across all patients and cohorts |
| 5 | Module 2 | BioClinicalBERT semantic embedding | Pretrained BioClinicalBERT as frozen feature extractor; [CLS] token embedding; PCA compression; seven NLP signatures |
| 6 | — | Feature normalization | Z-score standardization (training-set parameters); one-hot encoding; exclusion of high-missingness variables |
| 7 | Module 3 | Multimodal feature fusion | Late fusion via horizontal concatenation of imaging, NLP, and clinical feature groups |
| 8 | Module 3 | MLP classifier training | Multilayer perceptron with ReLU/sigmoid activations; dropout regularization; gradient-based optimization; binary cross-entropy loss |
| 9 | — | Model evaluation | Youden-index threshold; AUC with 95% CI; DeLong test; calibration curves; decision curve analysis |
| 10 | Module 4 | SHAP interpretability + clinical evaluation | Post hoc SHAP analysis; independent dual-reader human-machine comparison with and without AI assistance |

**S2.4 Reproducibility Statement**

This supplementary document describes the computational workflow, data flow, module functions, and quality control considerations for the multimodal MCE prediction framework presented in the main manuscript. Its purpose is to enhance the transparency and interpretability of the methods and to facilitate independent replication of the analytical approach.

The step-by-step workflow detailed in Section S2.2 reflects the actual sequence of operations implemented in the study, from data import through model evaluation and clinical interpretation. Each step specifies the input data, the processing operations performed, the output produced, and the quality control measures adopted to ensure validity and reproducibility.

The implementation used a Python-based deep learning environment for image processing, feature extraction, natural language processing, model training, and interpretability analysis, together with an R-based statistical computing environment for cohort description, performance evaluation, and decision curve analysis. All software environments, library dependencies, random seeds, data partition indices, normalization parameters, and evaluation scripts should be recorded and archived to enable exact reproduction of the reported results.

Investigators seeking to replicate or extend this work should ensure that their implementation faithfully follows the module sequence, data flow, and quality control procedures described herein. Any deviation in preprocessing, feature extraction, fusion strategy, or evaluation protocol may affect the comparability of results. The complete analysis pipeline, including version-controlled scripts, should be maintained and made available in accordance with institutional and journal data-sharing policies.
