# Supplementary material for: Malignant cerebral edema after endovascular thrombectomy: a multimodal prediction model based on post-thrombectomy cerebral hyperdensity and natural language processing
Source: Front Med (Lausanne). 2026 Aug 5;13:1857047. doi: 10.3389/fmed.2026.1857047 (PMC13485725; doi:10.3389/fmed.2026.1857047)
Supplement: Supplementary file 3 [file Table_3.docx]

**Supplementary Table S1 | Comparative performance of four convolutional neural network architectures for malignant cerebral edema prediction in the training and external test cohorts.**

| **Signature** | **Cohort** | **Accuracy** | **AUC** | **95% CI** | **Sensitivity** | **Specificity** | **PPV** | **NPV** | **Precision** | **Recall** | **F1** | **Threshold** |
| --- | --- | --- | --- | --- | --- | --- | --- | --- | --- | --- | --- | --- |
| VGG-11 | Train | 0.969 | 0.996 | 0.9919–1.0000 | 1.000 | 0.960 | 0.883 | 1.000 | 0.883 | 1.000 | 0.938 | 0.271 |
|  | Test | 0.628 | 0.501 | 0.3576–0.6453 | 0.409 | 0.703 | 0.321 | 0.776 | 0.321 | 0.409 | 0.360 | 0.245 |
| GoogLeNet | Train | 0.965 | 0.996 | 0.9901–1.0000 | 0.981 | 0.960 | 0.881 | 0.994 | 0.881 | 0.981 | 0.929 | 0.280 |
|  | Test | 0.663 | 0.577 | 0.4303–0.7232 | 0.591 | 0.687 | 0.394 | 0.830 | 0.394 | 0.591 | 0.473 | 0.161 |
| ResNet-50 | Train | 0.991 | 0.999 | 0.9967–1.0000 | 1.000 | 0.989 | 0.964 | 1.000 | 0.964 | 1.000 | 0.981 | 0.463 |
|  | Test | 0.721 | 0.661 | 0.5188–0.8023 | 0.545 | 0.781 | 0.462 | 0.833 | 0.462 | 0.545 | 0.500 | 0.314 |
| ResNet-101 | Train | 0.974 | 0.993 | 0.9839–1.0000 | 0.943 | 0.983 | 0.943 | 0.983 | 0.943 | 0.943 | 0.943 | 0.412 |
|  | Test | 0.779 | 0.707 | 0.5548–0.8599 | 0.636 | 0.828 | 0.560 | 0.869 | 0.560 | 0.636 | 0.596 | 0.354 |

All models were trained on a single representative non-contrast CT slice showing post-thrombectomy cerebral hyperdensity. The optimal classification threshold was determined by the Youden index on the training cohort and applied to the external test cohort. AUC, area under the receiver operating characteristic curve; CI, confidence interval; CNN, convolutional neural network; F1, F1 score; NPV, negative predictive value; PPV, positive predictive value; VGG, Visual Geometry Group network.
